# Geographical Distribution and Long-Term Monitoring of *Physokermes hellenicus* (Hemiptera: Coccomorpha: Coccidae) on *Abies* spp. (Pinales: Pinaceae) in Greece

**DOI:** 10.3390/insects12111001

**Published:** 2021-11-06

**Authors:** Iosif Papanastasiou, Nickolas G. Kavallieratos, Georgios Th. Papadoulis, Christina Emmanouil, Nikolaos G. Emmanouel

**Affiliations:** 1 Laboratory of Agricultural Zoology and Entomology, Department of Crop Science, Agricultural University of Athens, 75 Iera Odos Street, 11855 Athens, Greece; gpapadoulis@aua.gr (G.T.P.); ceaz2emn@aua.gr (N.G.E.); 2 Hellenic Agricultural Organization-DEMETER, 56–58 Kourtidou Street and Nirvana Street, 11145 Athens, Greece; 3 School of Spatial Planning and Development, Aristotle University of Thessaloniki, University Campus, 54124 Thessaloniki, Greece; chemmanouil@plandevel.auth.gr

**Keywords:** *Physokermes hellenicus*, latitudinal distribution, *Abies cephalonica*, abundance

## Abstract

**Simple Summary:**

The scale *Physokermes hellenicus* (Kozár & Gounari) (Hemiptera: Coccidae) benefits apiculture because it supplies bees with honeydew in Greek fir forests. However, there is limited literature available on its geographical distribution and level of infestation. Thus, in the current study, we investigated these issues in several mountains of Greece. Additionally, *P. hellenicus* infestation in combination with its natural enemies and honeydew production was monitored for a long period in three (Menalon, Parnis and Tymfristos) highland bee foraging areas. Overall, there was an extensive geographical distribution of the scale, which was negatively correlated with the latitude of the surveyed areas. A decline in *P. hellenicus* infestation in Menalon resulted in a reduction in honeydew production by this scale.

**Abstract:**

The scale *Physokermes hellenicus* (Kozár & Gounari) (Hemiptera: Coccidae) has been recently included in the Greek entomofauna as a beneficial honeydew species. However, there are no adequate data about its geographical distribution and degree of infestation. Therefore, a study was conducted to examine these parameters in fifteen mountains of Greece. Furthermore, the monitoring of *P. hellenicus* infestation was carried out over a six-year period with regard to natural enemies and honeydew presence at three mountains (i.e., Menalon, Parnis and Tymfristos) that are traditional honeybee foraging areas. An extensive geographical distribution of the scale was negatively correlated with the latitude. Over the period of the study, *P. hellenicus* infestation exhibited a decreasing trend in the three mountains, which was more obvious at Menalon. The abundance of natural enemies of *P. hellenicus*, their effectiveness on honeydew excretion and the fecundity of *P. hellenicus* are discussed. The reduction in the honey produced at the Menalon mountain (a protected designation of origin product) could be attributed to the reduced presence of *P. hellenicus* in the fir forest. Among the other identified arthropods, *Dreyfusia nordmannianae* Eckstein (Hemiptera: Adelgidae) is reported for the first time infesting *Abies cephalonica* (Pinales: Pinaceae) in Greece. Furthermore, this species is reported for the first time as a co-parasite with *P. hellenicus* on *A. cephalonica* in Greece. Since *D. nordmannianae* is a serious pest, additional research is needed to determine its status in Greek fir forest ecosystems.

## 1. Introduction

Over the last two decades, studies on scale insects (Hemiptera: Coccomorpha) of the Mediterranean basin have been intensified with remarkable findings [1,2,3,4,5]. In Greece, a total of 253 scale species have been identified so far [4]. However, many more species are expected to be discovered due to the diverse geographical terrain of Greece [2,4].

Female individuals of soft scale insects of the genus *Physokermes* (Hemiptera: Coccidae) resemble the axillary undeveloped shoots of plants commonly known as unarmored bud scale insects. They are herbivore oligophagous insects that settle either on foliage or on branches and infest tree species mainly of the genera *Abies*, *Pinus*, *Picea*, *Pseudotsuga* (Pinales: Pinaceae) and secondarily of the genera *Tsuga* and *Juniperus* (Pinales: Pinaceae) [6].

Genus *Physokermes* includes thirteen species, seven of which occur solely in the Palaearctic, four exclusively in the Nearctic region, one in both the Palaearctic and Nearctic and one species without a host record. *Physokermes hemicryphus* (Dalman) (Hemiptera: Coccidae) and *P. piceae* (Schrank) (Hemiptera: Coccidae) are the most cosmopolitan species as they have been recorded in 25 and 26 different countries, respectively [6,7,8,9]. In several countries such as Latvia, Lithuania, Serbia, Sweden and the USA, these insects are considered serious pests causing considerable primary damage on trees (i.e., reduction in shoot and needle growth, chlorosis and falling of leaves, drying of branches, partial or whole plant death). Furthermore, the production of sugary columnar excretions forms an ideal substrate for fungal growth, which can cause severe malfunction of photosynthesis and transpiration, decelerating the growth of infested plants [10,11,12,13,14,15]. Nevertheless, there are no data documenting that *Physokermes* spp. cause any growth malfunction to *Abies* spp. in Greece, even though they commonly occur on these tree species.

In contrast, *Physokermes* spp. constitute beneficial insects and are strongly connected with annual honey production in Greece [16]. Due to the construction of their mouthparts, they suck sap and excrete honeydew, which is used by bees to a large extent. Due to this valuable interaction, there is an increasing interest for data concerning new species of *Physokermes*, their distribution and natural enemies [9,16].

Hitherto, the studies dealing with the distribution of *Physokermes* spp. in Greek fir forests include four different species: *P. hellenicus* (Kozár & Gounari) (Hemiptera: Coccidae), *P. hemicryphus*, *P. inopinatus* (Danzig & Kozar) (Hemiptera: Coccidae) and *P. piceae*. According to Santas [17], *P. hemicryphus* has an extended distribution outspread in fir forests of Greece. The most frequent hosts are *Abies cephalonica* (Loudon) (Pinales: Pinaceae) and *A. borissi-regis* (Mattf.) (Pinales: Pinaceae). The author reported that this scale occurs on the mountains Ainos, Giona, Dirfis, Parnis, Parnon and Tymfristos as well as in the towns of Tripoli, Grevena and Lamia, on ornamental fir trees. *Physkermes piceae* has been recorded for the first the time by Santas [18], infesting fir trees of the genera *Abies* on the mountains of Giona and Parnassos. Many years later, the Hungarian spruce scale *P. inopinatus* was detected for the first time on *A. cephalonica* in a forest area of the Taygetus mountain (Peloponnese, southern Greece) [19]. Later, the new species *P. hellenicus* was found infesting *A. cephalonica* on mountains Ainos, Menalon and Panachaiko [9]. Recently, *P. hellenicus* was recorded on several mountains, i.e., Dirfys, Helmos, Heliconas, Kaliakouda, Parnis, Parnon, Taygetus, Parnon, Metsovo, Vardousia and Ziria [4,16,20], while *P. picea* was recorded on the Taygetus mountain [4]. Additionally, the genus *Juniperus* has been identified as a host plant of *P. hellenicus* on the Taygetus mountain [4].

In Greece, the honeydew honey from fir forests (*Abies* spp.) corresponds to 5–10% of the total annual production [9,18,21,22,23]. On the lush slopes of the mountain Menalon (Peloponnesus, southern Greece), honeybees mainly exploit the honeydews of *P. hemicryphus* in combination with excretions of the *Eulecanium sericeum* (Lindiger) (Hemiptera: Coccidae) and *Mindarus abietinus* (Koch) (Hemiptera: Aphididae) that infest native fir trees (*A. cephalonica*). The result of this interaction is the production of a honeydew honey that exhibits certain physicochemical characteristics [23,24]. This special fir honey, known as “Menalou Vanillia”, is one of the two types of honey that are officially recognized by Greek legislation as a product of protected designation of origin [25]. Its pearl–amber color, thick texture, buttery flavor and mild resinous aroma along with other special physicochemical parameters compose a unique product [23,26] that is highly acceptable by consumers. In the past, beekeepers believed that honey originating from the nectar of flowers is more attractive to consumers than the honey originating from the excreta of insects [27]. Today, this opinion has been altered in favor of honeydew honey instead of blossom honey due to the high antioxidant and antibacterial activity along with the great nutritional value of the former [28].

The occurrence of the recently described *P. hellenicus* was previously recorded in some locations mainly of southern Greece given with a short description of its biological cycle [4,9]. Later, information for the spectrum of natural enemies of *P. hellenicus* in Greece was provided [16]. Among *Physokermes* species, only *P. hemicryphus* has been studied for a ten-year period by Santas [17]. Based on the recent findings [16], we hypothesized that the presence of *P. hellenicus* is expanded in a larger geographical part of Greece. Therefore, the objective of the present study was to examine the relation between the infestation degree of fir nodes by *P. hellenicus* and its geographical distribution in Greece. Moreover, the infestation appearing at traditional honeybee forage areas on certain fir mountains of Greece has been monitored over a six-year period with regard to natural enemies and honeydew flow of *P. hellenicus*.

## 2. Materials and Methods

**Figure 1:**
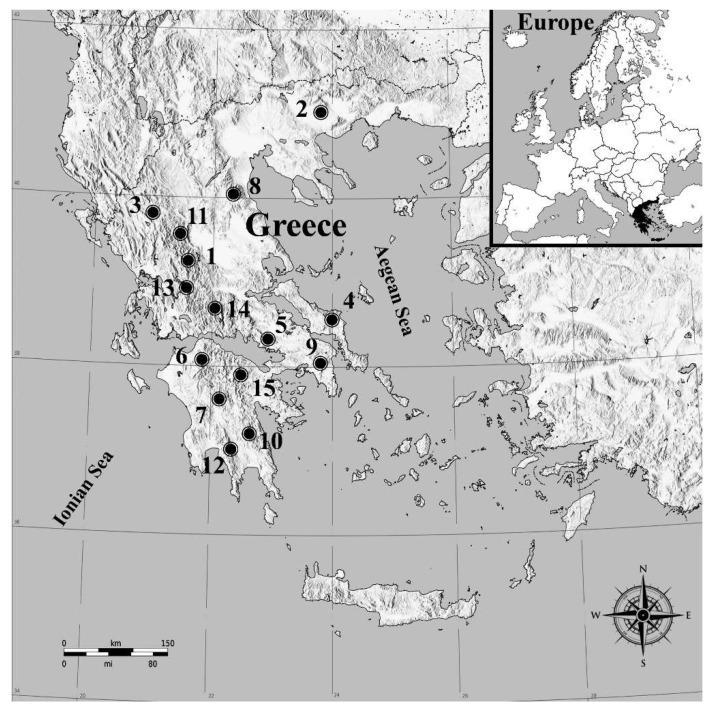
Map of Greece with the sampled mountains: Agrafa—1, Ano Vrontous—2, Athamanika—3, Dirfis—4, Helicon—5, Helmos—6, Menalon—7, Olympus—8, Parnis—9, Parnon—10, Pindos (central)—11, Taygetus—12, Tymfristos—13, Vardousia—14 and Ziria (Killini)—15.

In 2013, an extended field sampling was conducted to detect and confirm the presence of *P. hellenicus* in the Greek mountains. During the period from April to May, 153 samples were collected from fir (*Abies* spp.) forests located on fifteen different mountains of northern (Agrafa (5 samples), Ano Vrontous (3 samples), Athamanika (4 samples), Olympus (1 sample), Central Pindos (7 samples)), central (Dirfis (8 samples), Helicon (4 samples), Parnis (12 samples), Tymfristos (26 samples), Vardousia (5 samples)) and southern (Helmos (5 samples), Menalon (40 samples), Parnon (13 samples), Ziria (Killini) (14 samples), Taygetus (6 samples)) Greece (Figure 1). Details of each sampling point are given in Table 1. The selected sampling period is considered to be ideal because the exoskeletons of the collected female adults were not yet sclerotized and their taxonomic characters are sufficiently distinguishable [9]. Each sample consisted of two 4yr. terminal branches (25–30 cm in length) of one fir tree that was collected with a telescopic tree pruner at a height of up to 3 m above the ground. The samples were separately kept inside polypropylene bags, labeled and transferred to the laboratory. Each branch was carefully examined using a Stemi 2000-C (Zeiss, Göttingen, Germany) stereoscope and entomological forceps. When adult females of *P. hellenicus* were found, the sample was characterized as positive; otherwise it was marked as negative for infestation. In addition, from each positive sample three female adults were stored in 95% ethanol for slide preparations. Slide-mounted specimens were prepared according to a modified method of Ben Dov and Hodgson [29]. The morphological identification was performed with an Axiostar plus trinocular microscope (Zeiss, Göttingen, Germany) at a magnification of 400× by following the key of Kozár et al. [9].

Subsequently, in order to monitor the life cycle of *P. hellenicus*, a sampling period was conducted at three different mountains from 2013 to 2018 at Parnis and Menalon, while it was conducted at Tymfristos from 2014 to 2018. At Menalon, there were ten sampling points (MO2, MO5, MO8, MO9, MO11, MO14, MO19, MO28, MO29, MO36) ranging in altitude from 947 to 1472 m. At Parnis, there were three sampling points (PA1, PA5, PA7) ranging in altitude from 1163 to 1276 m, and at Tymfristos there were three sampling points (TS24, TS25, TS26) ranging in altitude from 912 to 1267 m. Differences in the number of sampling points among mountains were based on the fact that the sizes of fir vegetation were different (Menalon > Parnis = Tymfristos) These mountains (and their sampling points) were selected on the basis of the following criteria: (i) the presence of *P. hellenicus* scales; (ii) sampling from a range of different altitudes; (iii) the fact that areas around the sampling sites are representative forage areas for bees in southern and central Greece; (iv) our previous findings [16]. Only Parnis is excluded from the third criterion where honeybee colonies were forbidden temporarily for foraging due to a disastrous fire in 2007. The sampling period was held from January 2013 to December 2018. Samples were collected every 30 days in January, February, March, October, November and December and every 20 days in April, May, June, July, August and September.

The following parameters were recorded during stereoscopical observations in the laboratory: (a) the age and number of nodes of the fir branches; (b) the number of female individuals inside the node, their stage of life cycle, the presence of honeydew (three categories were set to evaluate honeydew quantity: absence of honeydew, one drop of honeydew behind the insect and many drops all over the insect and the node) and the period of honeydew flow; (c) the activity of natural enemies (monitored as suggested by Papanastasiou et al. [16]); (d) the presence/absence of male individuals alive on the foliage. Slide-mounted specimens were prepared and examined microscopically for the study of the life cycle. Finally, any other arthropod was collected for identification. During the survey, images were captured with a Nikon Coolpix 4500 digital camera (Tokyo, Japan).

To study the degree of infestation, the percentage of infestation [I_(%)_] of each positive sample was calculated on the basis of the formula: Ι_(%)_ = (Ν_Ι_ × 100)/Ν_Τ_, where Ν_Τ_ is the total number of nodes and Ν_Ι_ is the number of infested nodes with *P. hellenicus* individuals. To study the role of the latitude and altitude of the sampling point in the preference of the insect, a multiple linear regression analysis for infestation (dependent variable) against the latitude and altitude (independent variables) of sampling points was performed at a level of significance *a* = 0.05 using the extension XLSTAT Ver. 2021.2 from Microsoft Excel [30]. To study the degree of infestation [I_(%)_] at the three mountains, i.e., Menalon (6yr.), Parnis (6yr.) and Tymfristos (5yr.), a trend analysis was performed using MS Excel 2010.

To study the activity of natural enemies [E_(%)_] of the adult females, the percentage [E_(%)_] was calculated on the basis of the formula E_(%)_ = (F_E_ × 100)/F_T_. F_E_ is the number of adult females with natural enemies and F_T_ is the total number of adult females. Data on the activity of natural enemies were analyzed by using a two-way AΝOVA with the activity of natural enemies as the dependent variable. Mountain and natural enemies (i.e., parasitoids, predators) were the main effects. Healthy adult female individuals were also considered in the analysis. Means were separated by the Tukey–Kramer honestly significant difference (HSD) test at 0.05 probability [31] using JMP 14 software (SAS Institute Inc. Cary, NC, USA) [32]. Prior to the analysis, data were transformed according to arcsin of square root to normalize variances and standardize means [33]. To study the activity of natural enemies [E_(%)_] at the three mountains, i.e., Menalon (6yr.), Parnis (6yr.) and Tymfristos (5yr.), a trend analysis was performed using MS Excel 2010. 

To study the presence of honeydew of female individuals, the percentage [H_(%)_] was calculated on the basis of the formula H_(%)_ = (I_H_ × 100)/I_T_, where I_H_ is the number of female individuals with honeydew and I_T_ is the total number of female individuals. To study the presence of honeydew at the three mountains, i.e., Menalon (6yr.), Parnis (6yr.) and Tymfristos (5yr.), a trend analysis was performed using MS Excel.

## 3. Results

**Figure 2:**
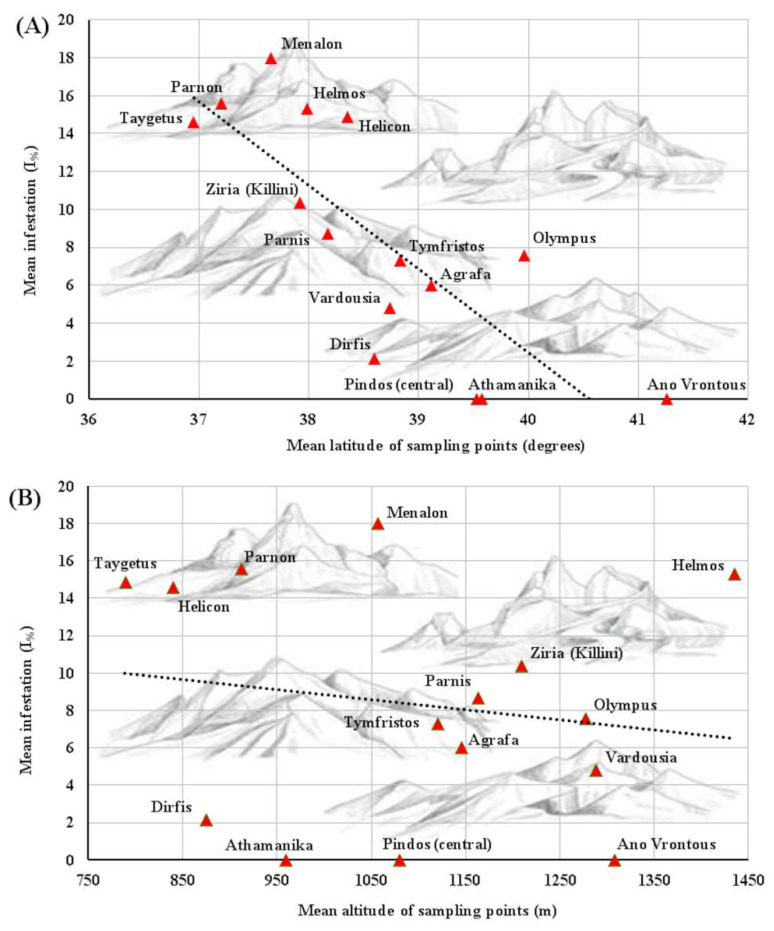
Percentage infestation [I_(%)_] of fir tree (*Abies* spp.) samples by *P. hellenicus*, per sampled mountain, against latitude (**A**) and altitude (**B**) of sampling points at different mountains of Greece.

On the basis of morphological identification [9], only *P. hellenicus* individuals were detected. The examination revealed 111 positive (72.5%) samples out of 153 samples regarding the presence of *P. hellenicus*. No infestation was recorded at the sampling points of mountains Athamanika, Ano Vrontous and Pindos (central). Among positive samples, the mean value of the percentage infestation [Ι_(%)_] ranged from 18% at the mountain Menalon to 2.2% at the mountain Dirfys. The highest and the lowest values (different than zero) were recorded at mountains Parnon (PS12: 57.6%) and Parnis (PA3: 0.8%), respectively (Table 1). The multiple linear regression analysis (Equation model: Infestation = 171.2 − 4.3 × latitude + 0.003 × altitude; *R*^2^ = 0.154) showed a statistically significant contribution of latitude (*p* < 0.0001), while altitude did not show any significance (*p* = 0.436) (Figure 2A,B).

During the monitoring of *P. hellenicus* life cycle at the three sampled mountains (Menalon, Parnis, and Tymfristos), the mean number of the examined nodes per year and per sampling point on the mountains Menalon, Parnon and Tymfristos was 746, 755 and 727, respectively. Their age ranged from the current year (0yr. old) up to 4yr. old. The majority of them (42.4%) belonged to the current year nodes followed by the 1yr. old (28%), the 2yr. old (16.8%), 3yr. old (8.8%) and the lowest (4%) to 4yr. old nodes.

The examination of the samples revealed the following stages of *P. hellenicus*. Inside the nodes: 1st instar larvae-crawlers (L1), 2nd instar female larvae (L2 ♀), 3rd instar female larvae (L3 ♀), transitional stage between 3rd instar female larvae and female adult (L3→adult ♀), female pre-reproductive adult (adult-pre ♀), female reproductive adult (adult ♀), matured eggs (eggs) and dead female adult of previous generation (adult old ♀). At the foliage: 1st instar larva-crawlers (L1), immature stages of male individuals (L2 ♂, prepupae ♂ and pupae ♂), adult male (adult ♂) and empty waxy covers of males (test). *Physokermes hellenicus* completed one generation per year on all sampled mountains. Hibernation was performed at L3 ♀ and L2 ♂ stages for the female and male individuals, respectively. Almost 70% of female individuals were found inside the current and 1yr. old nodes. Female larvae of the 2nd instar were the most rarely observed among all female developmental stages. The highest total number of female individuals (20,299) was observed at the mountain Menalon and the lower (1799) at mountain Tymfristos (Table 2).

During our study, the infestation [Ι_(%)_] of *P. hellenicus* reached very high values, i.e., 100% at Menalon and 95.2% at Parnis (Table 3.). However, when taking into account all the sampling points, the higher mean value of the infestation was observed in 2013 at mountains Parnis (37.8%) and Menalon (30.6%), while in 2015 it was observed at Tymfristos (11.5%).

**Figure 3:**
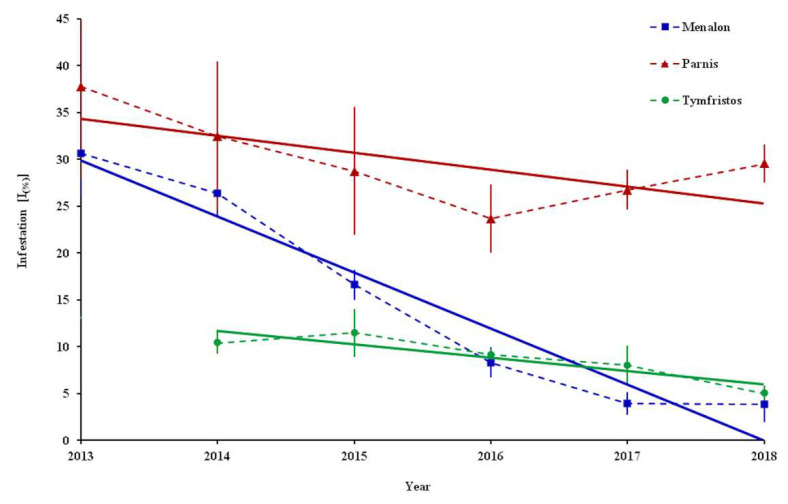
Percentage infestation [I_(%)_] (mean ± SE) of *Abies* spp. nodes by *P. hellenicus* during the sampling period 2013–2018 at Menalon and Parnis and 2014–2018 at Tymfristos. Red, blue and green lines indicate the trends of mean values of Menalon, Parnis and Tymfristos, respectively.

Interestingly, the infestation exhibited a decrease at Menalon and Tymfristos, reaching 3.8 and 5% in 2018, respectively. The analysis at the three mountains during the sampling period revealed a negative trend according to the estimated equation models in Menalon (infestation = −5.9879 × year + 12083; *R*^2^ = 0.9392), Parnis (infestation = −1.8073 × year + 3672.4; *R*^2^ = 0.4834) and Tymfristos (infestation = −1.4211 × year + 2873.8; *R*^2^ = 0.8179) (Figure 3).

**Figure 4:**
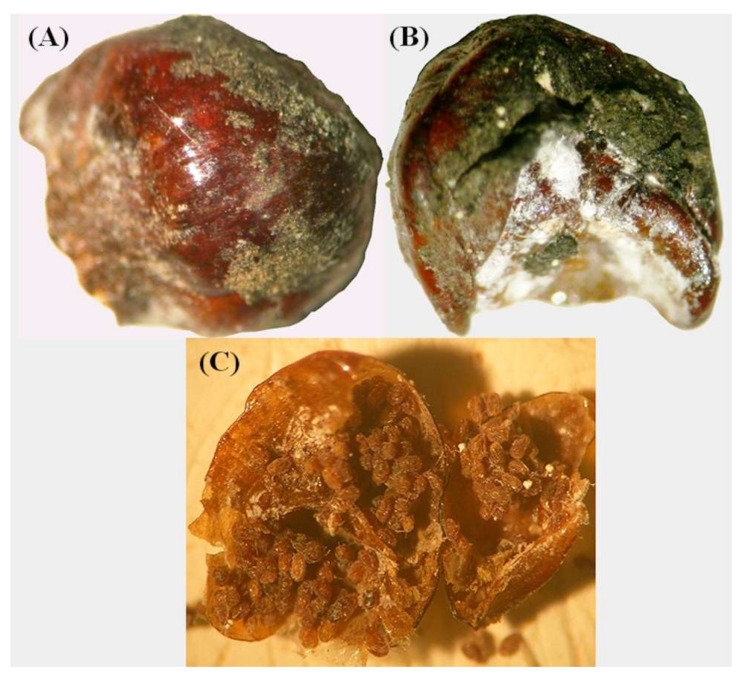
Dead-rotten adult female *P. hellenicus* with fungus on its surface: (**A**) dorsal view, (**B**) ventral view and (**C**) dead eggs of *P. hellenicus* inside the dead body of *P. hellenicus*.

**Figure 5:**
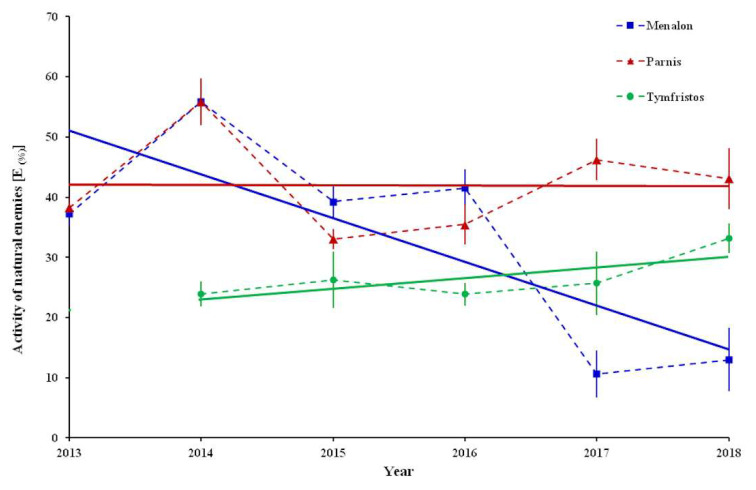
Activity of natural enemies [E_(%)_] (mean ± SE) of adult females of *P. hellenicus* during the sampling period 2013–2018 at Menalon and Parnis and 2014–2018 at Tymfristos. Red, blue and green lines indicate the trends of mean values of Menalon, Parnis and Tymfristos, respectively.

Observations on natural enemies revealed that their activity against female individuals of *P. hellenicus* was stereoscopically detectable from April to October. Furthermore, several adult ♀ individuals were covered with a grimy dark green to black soot outside, which resembled sooty mold fungus, and they were rotten inside, usually with dead eggs (Figure 4A–C). Although the cause of this situation was not defined, due to the considerable numbers observed, these individuals were counted and classified into an additional group labeled as “undefined”. The percentage of the activity of natural enemies [E_(%)_] of ♀ adults exhibited mean values that ranged from 10.7% (in 2017) to 55.9% (in 2014) at Menalon, from 33.0% (in 2015) to 55.8% (in 2014) at Parnis and from 23.9% (in 2016) to 33.2% (in 2018) at Tymfristos (Figure 5). The analysis in Menalon during the sampling period revealed a negative trend according to the equation model: activity of natural enemies = −7.2633 × year + 14,672; *R*^2^ = 0.5951, while at the other two mountains the patterns were different (Parnis: activity of natural enemies = −0.0604 × year + 163.62; *R*^2^ = 0.0002, Tymfristos: activity of natural enemies = −1.7816 × year − 3565.1; *R*^2^ = 0.5439) (Figure 5).

All main effects and the associated interactions were significant (Table 4). At Menalon, the parasitoid *Pseudorhopus testaceus* (Ratzeburg) (Hymenoptera: Encyrtidae) (14.0%), the predators (9.2%) and the “undefined” factor (11.3%) were significantly higher than the parasitoids *Anthribus fasciatus* Förster (Coleoptera: Anthibidae) (4.6%) and *Microterys lunatus* (Dalman) (Hymenoptera: Encyrtidae) (3.7%) (Table 5). At Parnis, *P. testaceus* (17.5%) was significantly higher than the other natural enemies. At Tymfristos, *P. testaceus* (10.2%) was significantly higher than *A. fasciatus* (0.5%) and *M. lunatus* (3.0%). *Anthribus fasciatus* was significantly lower at Tymfristos (0.5%) than at Parnis (6.4%) and at Menalon (4,6%). In total, the mean activity of natural enemies exhibited higher values at Menalon (42.8%) and Parnis (43.5%) than at Tymfristos (26.1%).

**Figure 6:**
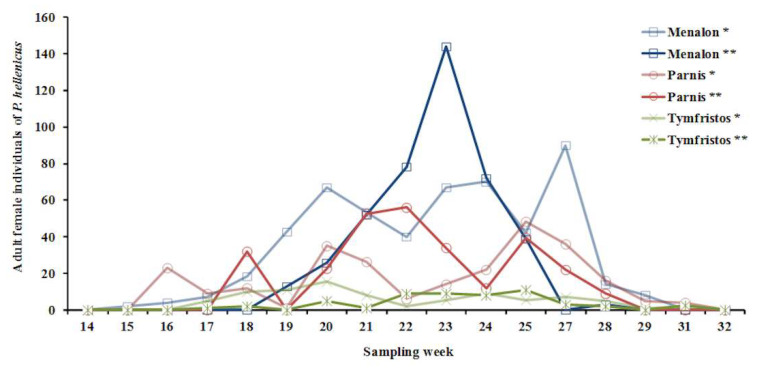
Mean value of the alive female adults of *P. hellenicus* inside nodes of *Abies* spp. during the entire sampling period at Menalon, Parnis and Tymfristos per week of the year and per amount of honeydew (* = one drop of honeydew behind the insect, ** = many drops diffusible all over the insect).

Honeydew production is mainly secreted by adult female individuals (96%), while the remaining (4%) amount is secreted by all the other stages (Table 6). The period of honeydew production lasted for about 14 weeks at Menalon, Parnis and Tymfristos. Initially, females excreted honeydew at low quantities (from 15th to 19th week of the year), which was then followed by an increase and reaching a peak (from 20th to 25th week of the year), and finally there was a decrease gradually to zero (from 26th to 31st week of the year) (Figure 6).

**Figure 7:**
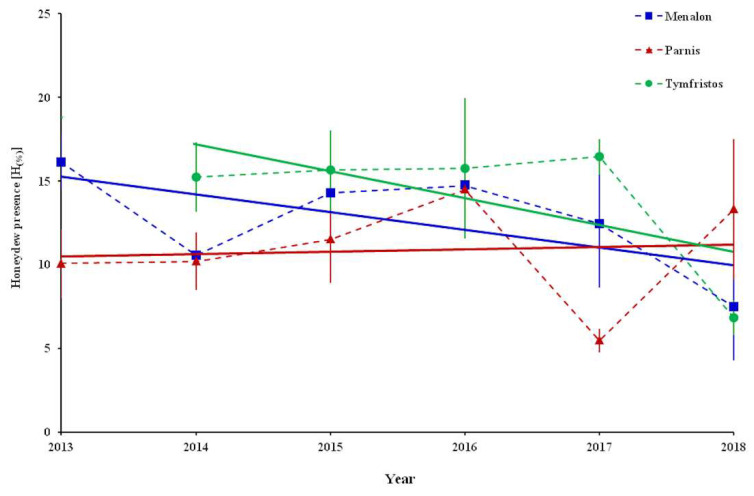
Percentage of honeydew presence [H_(%)_] of *P. hellenicus* female individuals during the sampling period 2013–2018 at Menalon and Parnis and 2014–2018 at Tymfristos. Red, blue and green lines indicate the trends of mean values of Menalon, Parnis and Tymfristos, respectively.

The lowest values of honeydew presence [H_(%)_] were recorded at Parnis (5.5%) in 2017 and at Menalon (7.5%) and Tymfristos (6.8%) in 2018. The analysis at the three mountains during the sampling period according to the equation models revealed a negative trend in Menalon (honeydew presence = −1.0576 × year + 2144.2; *R*^2^ = 0.39) and Timfrystos (honeydew presence = −1.5999 × year + 3239.3; *R*^2^ = 0.3956), while in Parnis no trend was observed (honeydew presence = 0.1415 × year − 274.28; *R*^2^ = 0.007) (Figure 7).

Natural enemies demonstrated a strongly negative impact on *P. hellenicus* honeydew production. On all mountains, the examined parasitized ♀ adults were found to produce lower honeydew amounts than the non-parasitized ones. In the case of *M. lunatus*, the percentage of ♀ adults with honeydew was extremely low (1.06%), while this percentage approached zero when ♀ adults were parasitized by *A. fasciatus* and the undefined factor. Furthermore, no honeydew was observed in the case of the presence of *P. testaceus* (Table 4).

Several arthropods were isolated from the collected fir tree samples. Nine of them were identified at species level while eleven were identified at genus level (Table 7). In addition, arthropods of the following orders were found: Diptera (Parnis), Psocoptera (Menalon, Parnis, Tymfristos and Parnon), Hemiptera of the family Ptininae (Parnis), Lepidoptera of the family Tortritidae (Menalon), Pseudoscorpionida (Parnis) and mites of the family Oribatidae (Menalon, Parnis, Tymfristos).

**Figure 8:**
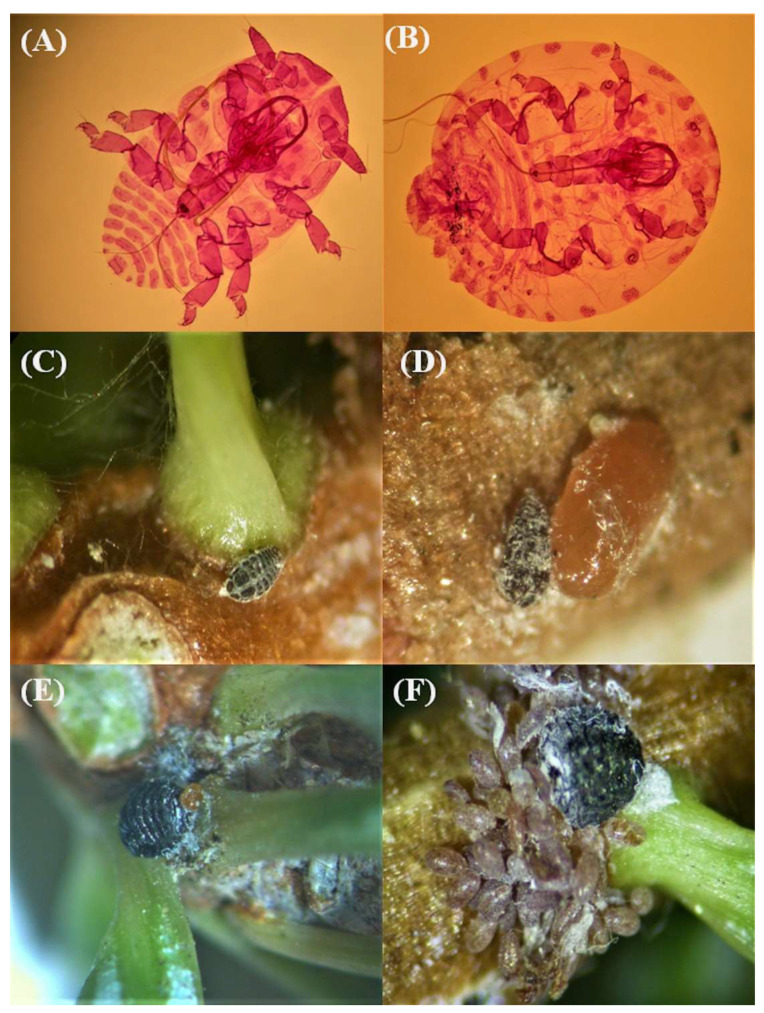
(**A**) Slide-mounted specimen of the 1st instar larva of the neosisten of *D. nordmannianae*, (**B**) slide-mounted specimen of mature larva of *D. nordmannianae*, (**C**) first instar larva of the overwintering neosisten of *D. nordmannianae*, (**D**) living mature larva of *D. nordmannianae* and *P. hellenicus* inside the node of *Abies* spp., (**E**) and (**F**) living mature larva of *D. nordmannianae* deposits clusters of brownish-orange eggs.

During the current survey, alive individuals of the silver fir wooly adelgid *Dreyfusia nordmannianae* Eckstein (Hemiptera: Adelgidae) were isolated for first time on *A. cephalonica* in Greece (Figure 8A,B), occurring in several survey sites of the different mountains (i.e., Menalon: MO8, MO9, MO11, MO14, MO28, MO29 and MO36; Parnis: PA1, PA5 and PA7; and Tymfristos: TS24, TS25 and TS27) and in all sampling years. Sistens were located on young needles and inside the nodes (Figure 8C). Occasionally, adelgid individuals were observed to live together inside the node with *P. hellenicus* (Figure 8D). Nymphs overwintered either inside fir nodes or in the base of fir needles. When they became mature, they started to deposit brown eggs in groups (Figure 8E,F).

## 4. Discussion

Our findings indicate that *P. hellenicus* was not detected only in Ano Vrontous, Athamanika and Pindos (northern Greece) out of the fifteen sampling mountains. Our extensive research in the Greek mountains revealed that in all positive sampling points, among *Physokermes* species, only *P. hellenicus* has been identified. This fact confirms the findings of our former study, which was conducted in fewer geographic areas [16]. However, it comes in contrast to the only previous study of *Physokermes* spp. across Greek mountains [17], where *P. hemicryphus* was found to be the most widespread species. Further comparison of our results with those of Santas [17] is not possible since no reference to slide-mounted specimens is provided. A newer survey on the scale insect fauna of Greece revealed that *P. hemicryphus* occurs in two areas of southern Greece, i.e., the mountain Taygetus on *Juniperus* sp. and Kalamata on *Abies* sp. [4]. Concerning *P. inopinatus*, there is only one record from the mountain Taygetus [19], which might be considered as a misidentification taking into account the recent data of the description of *P. hellenicus* (personal communication with Dr E. Szita). *Physokermes picea* on *Abies* sp. corresponds to a single record from mountains Parnassos and Giona in central Greece on fir trees [18] and to a recent record from the mountain Taygetus [4]. Although *P. hellenicus* is characterized as a Greek endemic species [2], it has also been reported to infest *Abies* spp. in forest and urban locations of Turkey [34,35,36].

The results of our study indicate a negative significant impact of latitude on the infestation level among the different sampled mountains. This finding could be attributed to the different environmental conditions between northern and southern areas. Indeed, the distribution of scale insects can be affected by climate variables (i.e., temperature seasonality, maximum temperature of the warm period, minimum temperature of the cold period, precipitation), vegetation, the structure of terrain and altitudes [37]. Northern latitudes consequently could adversely affect life stages, especially the crawlers, which are quite sensitive [38]. The highest number of female individuals has been observed on the 1-year-old nodes of the fir samples. More than half of the total number of female individuals has been detected on the current and 1-year-old nodes. Crawlers move robustly and settle towards the preferred feeding sites on young vegetation that has fully developed before the dispersal period [39]. Among female stages, the most rarely observed is the 2nd instar female larva. A similar observation has also been recorded in Ankara (Turkey) [35].

The fecundity of *P. hellenicus* exhibits variable ranging patterns as it has been previously reported from Greece, i.e., 100 to 230 eggs [9], and Turkey, i.e., 41 to 273 eggs [35]. However, there is no reference whether fecundity is affected by the activity of natural enemies. In a previous study, Papanastasiou et al. [16] documented that *P. testaceus* and *A. fasciatus* are able to suppress the reproductive potential of *P. hellenicus* in Greece. Moreover, the introduction of the relative species *Anthribus nebulosus* Förster (Coleoptera: Anthribidae) in Virginia (USA) caused a reduction in the populations of *P. inopinatus* and *P. piceae* [40]. In the present study, *P. testaceus* appeared to be the most abundant natural enemy of *P. hellenicus* in Greece, as reported in our previous study [16]. Additionally, *P. testaceus* constitutes an important natural enemy of other *Physokermes* species such as *P. hemicryphus* in Greece and Serbia [15,17] and *P. piceae* in Serbia [12,41]. Recently, a trophic interaction among *P. hemicryphus*, *P. testaceus* and *A. nebulosus* has been described in Serbia [41], a relation that should be also investigated in the case of *P. hellenicus*.

As it is indicated from the trend analysis, a gradual decrease in the infestation of fir nodes by *P. hellenicus* was recorded on Menalon. A similar reduction had been observed on Parnis until 2016, followed by a small increase over the next two years. At these two mountains, the maximum activity of natural enemies on female individuals in 2014 reached 56% of *P. hellenicus* individuals. On Tymfristos, the infestation exhibited a mild reduction from 2015 to 2018, where the activity of natural enemies did not exceed 34% of *P. hellenicus* individuals. Parasitoids, predators and entomopathogenic fungi in combination with abiotic (climatic) conditions are crucial factors that can regulate soft scale populations [42,43]. Although in the present study no climatic data were collected, it seems that the synthesis of the spectrum of natural enemies led to an essential decrease in the numbers of *P. hellenicus* in the investigated areas, especially on the mountain Menalon. A similar reduction in the population of *P. hemicryphus* was recorded due to the activity of natural enemies during an 8-year monitoring period in Greek mountains [17].

Scale insects can cohabit with microorganisms such as bacteria and fungi that improve their metabolic capacities, cover their certain diet requirements and make them resistant against life stresses, i.e., insect foes, toxic plant compounds and high temperatures [44]. Nevertheless, there are microorganisms, such as fungi, that can be lethal since they cause dramatic changes to the microbiota of scales [45]. In the present study, numerous adult female individuals of *P. hellenicus* (categorized as undefined) were found dead and partially covered by a dark green (fungus-like) microorganism, as in the case of wax scale insect *Ericerus pela* (Chavannes) (Hemiptera: Coccidae) [45]. Further studies are necessary to clarify the cause of this important finding, which could be useful for the improvement of our knowledge on natural enemies and their interactions as well as for the development of biological control programs against scale insects.

For a prolific honeydew flow, which can vary from year to year, a large population of the involved insects is needed [46]. Apparently, the vitality and the abundance of *P. hellenicus* are important for the presence of honeydew. The majority of the adult females of *P. hellenicus* infested by natural enemies were unable to produce honeydew drops, with the exception of those that were infested by *M. lunatus* [16]. Our results showed a reduction in the honeydew presence that may be attributed to the activity of natural enemies. Although these results can support the low formation of the fir honey “Menalou Vanillia” in the last few years in Greece [16], further investigation is considered necessary to clarify this issue. The main species that interfered with the production of this special type of honeydew honey is *P. hellenicus* rather than the relative *P. hemicryphus*, as was previously thought [9,17,22,23,24]. The lower *P. hellenicus* infestation of fir trees on the mountain Tymfristos suggests their limited contribution to the production of fir honeydew honey in central Greece. Furthermore, the detection of other important honeydew insects, such as *E. sericeum*, *Cinara pectinatae* (Nördl.) (Hemiptera: Aphididae), *Marchalina hellenica* (Gennadius) (Hemiptera: Margarodidae) and *M. abietinus*, in Greek mountains indicates their involvement in the production of fir honey, but without their contribution being known due to the lack of available published data. Interestingly, the insects related to honeydew honey can leave their genetic fingerprints inside honey [47]. Moreover, certain sugars, amino acids and inorganic ions contained in honey can be suitable markers for distinguishing the honeydew honeys derived from botanical (involving plants) and zoological (involving honeydew insects) origins [48]. Combined analyses of the DNA of honeydew insects that is included in various types of honey with the chemical composition of these types may assist in shedding light on their authentication credits.

Unlike many European countries, there are no published studies available regarding the potential adverse effects on the growth and health of fir trees infested by honeydew scale insects in forest areas of Greece. It seems that their natural enemies exert efficient pressure and maintain their population at a non-devastating level. Apparently, fir trees exist in harmony with *Physokermes* spp. in Greek forests, an issue that contributes significantly to the nutrition of honeybees.

One other outcome of our study that is worth noting was the identification of *D. nordmannianae* for first time on *A. cephalonica* at several sampling points. This record provides evidence of a possible extended distribution of *D. nordmannianae* in Greek mountains, but its population density still remains unknown. As far as we know, the distribution of *D. nordmannianae* covers areas from 52° N in the north (Poland) to 40° N in the south (northern border of Greece) and from 5° E in the west (western Alps) to 27° E in the east (Romania, Bulgaria) [49]. This tiny cryptic aphid is a serious pest of the Nordmann fir *Abies nordmanniana* (Steven) Spach (Pinales: Pinaceae), which is the main cultivated tree for Christmas in Central and Northern Europe [50], leading to severe losses in plantations [51,52,53]. Usually, *D. nordmannianae* infestation causes disorders in the whole plant, especially in stems and leaves [49]. The fact that *D. nordmannianae* has no parasitoids in nature [50] should not be overlooked since an outbreak may alter the balance of forest ecosystems in the Greek mountains.

## 5. Conclusions

To conclude, the results of this study revealed that the scale *P. hellenicus* is the most widespread among *Physokermes* species found on fir forests in Greece. Based on this exhaustive investigation at several mountains of southern, central and northern Greece, it becomes evident that climate differences associated with latitude have a significant negative effect on the infestation of fir nodes by *P. hellenicus*. Among the observed natural enemies, the polyembryonic wasp *P. testaceus* was the most abundant in Greece. Additionally, numerous adult female individuals of *P. hellenicus* with dead eggs covered with a fungus-like microorganism have been described for the first time. Further research is needed to enrich our knowledge on natural enemies. Furthermore, the aphid *D. nordmannianae* has been reported for the first time in Greece co-parasitizing *A. cephalonica* with *P. hellenicus*. As *D. nordmannianae* is a serious pest of fir trees, additional research is needed to determine its population density on Greek fir forest ecosystems. Our results suggest that the reduction in the protected designation of origin honey produced in Menalon could be attributed to the low observed *P. hellenicus* infestation of fir trees in that area.

## Figures and Tables

**Table 1:** Coordinates of sampling points and percentage infestation [I_(%)_] of *Abies* spp. samples by *P. hellenicus*.

Sampled Mountain	Sampling Code	Latitude	Longitude	Altitude (m)	Infestation (%)
1. Agrafa	AG1	N 39°04′04.2″	E 021°32′50.7″	1145	1.3
	AG2	N 39°06′06.4″	E 021°36′40.7″	715	7.2
	AG3	N 39°08′47.1″	E 021°36′48.8″	856	14.9
	AG4	N 39°07′50.3″	E 021°38′40.1″	879	4.3
	AG5	N 39°08′13.9″	E 021°40′13.3″	1371	2.3
2. Ano Vrontous	VS1	N 41°16′32.4″	E 023°38′55.9″	1307	0
	VS2	N 41°15′56.8″	E 023°39′18.7″	1318	0
	VS3	N 41°15′33.7″	E 023°39′11.8″	1295	0
3. Athamanika	AA1	N 39°35′01.1″	E 021°02′30.4″	959	0
	AA2	N 39°34′58.4″	E 021°02′06.3″	975	0
	AA3	N 39°34′56.6″	E 021°01′47.4″	998	0
	AA4	N 39°34′52.2″	E 021°01′18.2″	1088	0
4. Dirfis	DS1	N 38°36′03.3″	E 023°51′09.7″	874	0
	DS2	N 38°36′01.9″	E 023°51′41.9″	940	0
	DS3	N 38°35′55.0″	E 023°51′50.8″	953	0
	DS4	N 38°35′59.7″	E 023°52′09.0″	1012	0
	DS5	N 38°36′12.6″	E 023°52′16.1″	980	0
	DS6	N 38°35′55.8″	E 023°52′21.2″	923	0
	DS7	N 38°35′56.4″	E 023°52′46.2″	834	0
	DS8	N 38°35′55.8″	E 023°52′40.1″	867	17.2
5. Helicon	ES1	N 38°23′35.6″	E 022°49′53.7″	792	11.4
	ES2	N 38°22′17.5″	E 022°47′40.9″	910	20.7
	ES3	N 38°19′27.8″	E 022°51′05.5″	977	11.8
	ES4	N 38°19′15.9″	E 022°52′15.4″	902	15.5
6. Helmos	HS1	N 38°01′00.8″	E 022°10′31.7″	1434	23.0
	HS2	N 37°59′25.7″	E 022°08′37.3″	1155	21.3
	HS3	N 38°00′26.1″	E 022°08′22.3″	1230	12.5
	HS4	N 37°53′55.5″	E 022°11′51.6″	1226	2.0
	HS5	N 38°00′47.5″	E 022°09′27.0″	1192	17.8
7. Menalon	MO1	N 37°34′38.7″	E 022°17′19.9″	1056	0.8
	MO2	N 37°34′23.1″	E 022°16′42.7″	947	6.9
	MO3	N 37°35′38.5″	E 022°14′09.1″	1053	6.1
	MO4	N 37°36′19.5″	E 022°13′30.6″	1104	1.6
	MO5	N 37°37′15.6″	E 022°13′27.3″	1139	24.1
	MO6	N 37°37′46.8″	E 022°12′56.1″	1164	22.0
	MO7	N 37°38′03.9″	E 022°12′41.7″	1281	20.4
	MO8	N 37°38′24.7″	E 022°12′25.1″	1317	46.2
	MO9	N 37°38′57.4″	E 022°12′03.4″	1241	27.2
	MO10	N 37°39′38.2″	E022°11′57.3″	1175	13.4
	MO11	N 37°40′31,6″	E022°12′21.4″	1174	32.1
	MO12	N 37°40′58.5″	E 022°12′32.0″	1277	10.4
	MO13	N 37°40′39.5″	E 022°13′16.2″	1409	6.0
	MO14	N 37°40′24.4″	E022°14′00.4″	1472	16.8
	MO15	N 37°39′53.4″	E 022°15′06.3″	1557	18.8
	MO16	N 37°38′43.3″	E 022°16′03.1″	1542	6.6
	MO17	N 37°37′51.0″	E 022°16′11.1″	1404	18.2
	MO18	N 37°37′37.9″	E 022°15′53.7″	1295	20.0
	MO19	N 37°37′28.9″	E 022°16′49.8″	1129	12.9
	MO20	N 37°37′47.2″	E 022°18′08.1″	939	0.0
	MO21	N 37°32′58.2″	E 022°11′56.0″	1143	0.0
	MO22	N 37°33′34.9″	E 022°11′09.4″	1304	2.1
	MO23	N 37°34′01.2″	E 022°10′15.1″	1339	2.1
	MO24	N 37°34′39.3″	E 022°09′47.2″	1242	0
	MO25	N 37°35′59.7″	E 022°09′36.4″	1202	5.7
	MO26	N 37°36′43.3″	E 022°09′18.6″	1142	36.6
	MO27	N 37°37′12.6″	E 022°08′37.3″	1174	20.0
	MO28	N 37°37′37.3″	E 022°09′20.8″	1145	20.6
	MO29	N 37°38′35.3″	E 022°09′46.0″	1002	26.3
	MO30	N 37°39′13.9″	E 022°09′36.4″	1004	15.6
	MO31	N 37°39′48.8″	E 022°07′57.5″	1121	26.2
	MO32	N 37°40′41.2″	E 022°07′06.1″	1265	36.2
	MO33	N 37°41′26.4″	E 022°06′33.8″	1219	33.5
	MO34	N 37°42′08.3″	E 022°06′31.3″	1162	9.5
	MO35	N 37°39′35.3″	E 022°06′52.7″	1133	45.6
	MO36	N 38°54′23.4″	E 021°54′18.5″	1136	35.2
	MO37	N 37°40′03.1″	E 022°07′40.3″	1109	33.6
	MO38	N 37°42′02.7″	E 022°06′06.7″	1103	32.0
	MO39	N 37°42′03.9″	E 022°08′13.9″	1183	4.0
	MO40	N 37°47′16.4″	E 022°15′05.8″	782	25.0
8. Olympus	OS1	N 39°58′09.4″	E 022°31′18.2″	1277	7.6
9. Parnis	PA1	N 38°10′10.3″	E 023°43′27.9″	1163	14.5
	PA2	N 38°10′06.1″	E 023°43′58.9″	1187	10.3
	PA3	N 38°10′21.5″	E 023°44′29.4″	1140	0.8
	PA4	N 38°10′44.4″	E 023°44′11.5″	1045	7.0
	PA5	Ν 38°10′13.3″	E 023°44′11.6″	1195	31.2
	PA6	N 38°10′28.2″	E 023°43′54.9″	1243	0
	PA7	N 38°10′22.1″	E 023°43′52.0″	1276	20.2
	PA8	N 38°10′51.0″	E 023°43′42.1″	1063	7.9
	PA9	N 38°10′49.0″	E 023°43′02.8″	1115	4.3
	PA10	N 38°10′42.9″	E 023°42′33.6″	1119	6.9
	PA11	N 38°10′32.1″	Ε 023°42′02.5″	1081	1.5
	PA12	N 38°09′57.7″	E 023°42′08.2″	1020	0
10. Parnon	PS1	N 37°11′06.0″	E 022°32′34.6″	912	4.8
	PS2	N 37°11′17.6″	E 022°33′26.3″	1023	13.5
	PS3	N 37°11′28.5″	E 022°34′25.8″	1008	0.0
	PS4	N 37°11′07.7″	E 022°34′45.2″	1082	24.2
	PS5	N 37°10′16.1″	E 022°35′47.7″	1080	3.1
	PS6	N 37°10′02.7″	E 022°35′57.7″	1113	8.1
	PS7	N 37°09′43.5″	E 022°35′56.9″	1212	5.9
	PS8	N 37°11′55.2″	E 022°34′32.9″	1100	7.2
	PS9	N 37°12′43.2″	E 022°34′16.2″	1243	10.4
	PS10	N 37°12′53.6″	E 022°34′25.1″	1216	29.6
	PS11	N 37°13′27.1″	E 022°33′50.6″	1148	12.3
	PS12	N 37°19′40.7″	E 022°34′44.8″	871	57.6
	PS13	N 37°19′53.2″	E 022°35′15.2″	914	26.0
11. Pindos (central)	PI1	N 39°30′22.7″	E 021°32′25.5″	1079	0
	PI2	N 39°32′46.7″	E 021°28′15.3″	1215	0 *
	PI3	N 39°32′24.6″	E 021°28′12.5″	1191	0
	PI4	N 39°36′45.4″	E 021°29′45.5″	982	0
	PI5	N 39°30′52.7″	E 021°30′06.5″	1204	0 *
	PI6	N 39°31′11.6″	E 021°26′38.6″	1064	0
	PI7	N 39°29′45.4″	E 021°32′37.2″	974	0
12. Taygetus	TG1	N 36°58′03.2″	E 022°23′55.5″	839	0
	TG2	N 36°58′01.3″	E 022°23′17.5″	904	10.2
	TG3	N 36°57′50.6″	E 022°22′54.1″	1053	0
	TG4	N 36°57′34.7″	E 022°23′17.4″	1215	0
	TG5	N 36°57′13.1″	E 022°22′57.1″	1254	47.6
	TG6	N 36°53′51.8″	E 022°19′17.9″	1408	29.8
13. Tymfristos	TS1	N 38°54′35.0″	E 021°54′47.8″	906	13.8
	TS2	N 38°54′23.4″	E 021°54′18.5″	1025	4.6
	TS3	N 38°53′44.0″	E 021°53′16.1″	1146	0.0
	TS4	N 38°53′31.0″	E 021°52′36.9″	1158	1.5
	TS5	N 38°53′30.2″	E 021°52′18.8″	1045	0.0
	TS6	N 38°54′03.0″	E 021°50′34.7″	954	9.8
	TS7	N 38°53′35.8″	E 021°45′52.5″	790	2.7
	TS8	N 38°52′09.9″	E 021°45′05.9″	717	4.5
	TS9	N 38°50′11.5″	E 021°43′41.0″	816	4.7
	TS10	N 38°44′17.9″	E 021°39′09.9″	837	2.5
	TS11	N 38°44′00.8″	E 021°39′06.6″	803	8.1
	TS12	N 38°44′51.7″	E 021°38′47.2″	962	5.9
	TS13	N 38°45′17.1″	E 021°38′38.5″	1093	21.5
	TS14	N 38°44′34.4″	E 021°38′15.6″	1287	5.5
	TS15	N 38°44′17.6″	E 021°38′29.5″	1355	21.4
	TS16	N 38°52′55.8″	E 021°52′44.1″	1292	0.0
	TS17	N 38°52′05.5″	E 021°52′19.8″	1359	0.0
	TS18	N 38°51′52.1″	E 021°51′24.5″	1418	0.0
	TS19	N 38°51′18.4″	E 021°51′00.5″	1542	1.2
	TS20	N 38°50′24.6″	E 021°50′19.6″	1464	2.2
	TS21	N 38°49′28.0″	E 021°50′25.5″	1456	10.5
	TS22	N 38°48′25.3″	E 021°50′09.2″	1407	3.9
	TS23	N 38°48′03.3″	E 021°50′50.1″	1202	14.3
	TS24	N 38°50′28.8″	E 021°43′33.1″	912	12.9
	TS25	N 38°52′38.3″	E 021°47′24.0″	1042	15.2
	TS26	N 38°49′08.3″	E 021°49′47.5″	1267	22.6
14. Vardousia	VA1	N 38°41′47.1″	E 022°01′46.2″	1288	0
	VA2	N 38°42′17.3″	E 022°01′54.2″	1171	0
	VA3	N 38°42′57.9″	E 022°01′19.9″	1134	0
	VA4	N 38°43′18.3″	E 022°00′55.9″	830	0
	VA5	N 38°53′03.2″	E 022°03′04.2″	782	24.1
15. Ziria (Killini)	ZA1	N 38°01′11.3″	E 022°24′12.0″	1209	21.9
	ZA2	N 38°00′12.8″	E 022°23′33.4″	1075	8.2
	ZA3	N 37°59′39.3″	E 022°22′21.7″	953	20.3
	ZA4	N 37°55′50.2″	E 022°16′49.6″	883	9.7
	ZA5	N 37°55′35.8″	E 022°17′00.9″	883	3.9
	ZA6	N 37°55′31.4″	E 022°17′16.2″	883	0
	ZA7	N 37°52′37.3″	E 022°21′26.7″	986	0
	ZA8	N 37°52′05.3″	E 022°21′53.1″	1045	20.7
	ZA9	N 37°51′57.7″	E 022°21′56.2″	1063	23.7
	ZA10	N 37°51′44.3″	E 022°22′56.5″	855	0
	ZA11	N 37°51′31.8″	E 022°22′59.2″	741	22.1
	ZA12	N 37°51′29.8″	E 022°15′01.0″	1175	7.2
	ZA13	N 37°58′53.8″	E 022°27′50.0″	1132	4.8
	ZA14	N 37°58′42.0″	E 022°34′07.4″	931	2.8

An asterisk (*) indicates those sampling points where only dead adults of *P. hellenicus* were found.

**Table 2:** Total number of nodes of *Abies* spp. per age class, and female individuals of *P. hellenicus* contained in nodes at mountains Menalon, Parnis and Tymfristos during the entire experimental period.

		Class of Age Nodes				
Sampled Mountain	Nodes/Female Individuals	0	1	2	3	4
Menalon	nodes	18,722	12,414	7674	4112	1858
	female individuals	5595	8074	4232	1825	573
Parnis	nodes	5614	3942	2492	1347	559
	female individuals	4731	5283	2803	1203	392
Tymfristos	nodes	4929	3071	1691	812	406
	female individuals	678	815	237	62	7

**Table 3:** Maximum values of percentage infestation [I_(%)_] of *Abies* spp. nodes by *P. hellenicus*.

Sampling Points	Sampling Years					
	2013	2014	2015	2016	2017	2018
MO2	26.0	23.1	12.3	11.1	0.0	0.0
MO29	57.4	50.0	45.1	19.5	34.3	5.3
MO19	23.4	25.0	17.3	9.1	10.6	0.0
MO36	37.3	60.0	68.1	55.0	9.5	21.3
MO5	36.7	36.4	19.6	12.8	0.0	0.0
MO28	64.8	70.8	24.6	7.1	0.0	0.0
MO11	86.3	90.0	64.0	40.6	11.4	21.2
MO9	87.9	82.8	45.7	20.9	14.6	10.3
MO8	100.0	75.0	30.4	51.9	2.4	4.1
MO14	59.2	48.9	52.6	41.7	13.7	32.1
PA1	29.5	23.0	22.6	26.1	34.9	53.6
PA5	95.2	76.9	46.9	48.9	53.3	51.5
PA7	91.8	81.0	50.0	37.0	51.3	84.6
TS24	-	22.9	19.7	21.3	13.5	12.1
TS25	-	12.5	22.0	14.9	13.1	6.1
TS26	-	25.0	41.3	14.0	17.1	10.0

Where dashes exist no material was collected.

**Table 4:** ANOVA parameters of main effects and associated interaction for the activity of natural enemies [E_(%)_] of *P. hellenicus* in three mountains of Greece (error DF = 78).

Source	DF	*F*	*p*
Mountain	2	4.1	0.0211
Natural enemies	5	393.2	<0.0001
Mountain x natural enemies	10	9.1	<0.0001

**Table 5:** Mean values (±SE) of the activity of natural enemies [E_(%)_] of *P. hellenicus* in three mountains of Greece.

		Mountain			
Natural Enemies	Menalon	Parnis	Tymfristos	*F*	*p*
Healthy	57.2 ± 1.8 Ab	56.5 ± 2.6 Ab	73.9 ± 2.9 Aa	13.3	0.0007
*A. fasciatus*	4.6 ± 0.5 Da	6.4 ± 1.0 Ca	0.5 ± 0.5 Db	18.6	0.0002
*M. lunatus*	3.7 ± 0.5 D	5.4 ± 1.2 C	3.0 ± 0.4 C	2.0	0.1797 NS
*P. testaceus*	14.0 ± 1.1 Bab	17.5 ± 0.1 Ba	10.2 ± 0.6 Bb	4.6	0.0308
Predators	9.2 ± 1.0 C	8.3 ± 2.1 C	7.1 ± 1.6 BC	0.6	0.5499 NS
Undefined	11.3 ± 1.0 BCa	5.9 ± 0.7 Cb	5.3 ± 0.6 BCb	11.0	0.0016
*F*	245.3	103.1	172.4		
*p*	<0.0001	<0.0001	<0.0001		

Within each column, means followed by the same uppercase letters do not differ significantly, for Menalon DF = 5, 59, for Parnis DF = 5,17, for Tymfristos DF = 5, 17; Tukey–Kramer HSD test at 0.05. Within each row, means followed by the same lowercase letters do not differ significantly, in all cases for DF = 2, 15; Tukey–Kramer HSD test at 0.05. Where no letters exist, no significant differences were noted (NS).

**Table 6:** Number of *P. hellenicus* female individuals with honeydew at a parasitized and non-parasitized (healthy) status in three sampled mountains of Greece during the whole sampling period (2013–2018).

	Status	Healthy			*A. fasciatus*			*M. lunatus*			Undefined		
	Mountain	M	P	T	M	P	T	M	P	T	M	P	T
Life stage	L1	24 *	0	0	0	0	0	0	0	0	0	0	0
	L2 ♀	11 *	0	0	0	0	0	0	0	0	0	0	0
	L3 ♀	61 *	2 *	7 *	0	0	0	0	0	0	0	0	0
	L3→adult ♀	31 *	0	0	0	0	0	0	0	0	0	0	0
	adult-pre ♀	30 *	2 *	0	0	0	0	0	0	0	0	0	0
	adult ♀	1050 *	547 *	113 *	0	2 *	0	11 *	21 *	1 *	2 *	1 *	0
		829 **	460 **	73 **	0	0	0	0	0	0	0	0	0

The letters M, P and T correspond to mountains Menalon, Parnis and Tymfristos, respectively. The asterisks indicate the amount of honeydew (* = one drop of honeydew behind the insect, ** = many drops diffusible all over the insect).

**Table 7:** Arthropods found on fir samples of south and central Greece.

Species	Sampled Mountain
Coleoptera	
*Phalacrus* sp. (Phalacridae)	7
*Protapion* sp. (Apionidae)	7
*Otiorrynchus raucus* (Fabricius) (Curculionidae)	7
*Sitona* sp. (Curculionidae)	12
*Harmonia* sp. (Coccinellidae)	12
*Altica* sp. (Chrysomelidae)	7
Dermaptera	
*Forficula* sp. (Forficulidae)	6, 7, 9, 13
Hemiptera	
*Coreus marginatus* (L.) (Coreidae)	12
*Cinara pectinatae*	13
*Dinaspidiotus abietis* (Schrank) (Diaspididae)	7, 9, 12
*Dinaspidiotus abieticola* (Coroneos) (Diaspididae)	7, 9, 12
*Dreyfusia nordmannianae*	7, 9, 13
*Eulecanium sericeum*	7, 9, 10, 12, 13
*Marchlina hellenica*	6, 7, 9
*Mindarus abietinus*	5, 7, 9, 10, 12, 13
*Lepidosaphes* sp. (Diaspididae)	7, 9
*Leucaspis* sp. (Diaspididae)	7, 9
*Pseudococcus* sp. (Pseudoccocidae)	7, 9
Lepidoptera	
*Eupithecia* sp. (Geometridae)	9
Neuroptera	
*Chrysoperla* sp.(Chrysopidae)	9, 12

Numbers 5, 6, 7, 9, 10, 12 and 13 correspond to sampled mountains Helicon, Helmos, Menalon, Parnis, Parnon, Taygetus and Tymfristos, respectively.

## Data Availability

Data are contained within the article.
